# How many people must die from pandemics before the world learns?

**DOI:** 10.1002/gch2.1011

**Published:** 2016-11-15

**Authors:** Steven J. Hoffman

**Affiliations:** ^1^ Global Strategy Lab, Centre for Health Law, Policy & Ethics, Faculty of Law University of Ottawa Ottawa Ontario Canada; ^2^ Department of Global Health & Population, Harvard T.H. Chan School of Public Health Harvard University Boston Massachusetts USA; ^3^ Department of Clinical Epidemiology & Biostatistics and McMaster Health Forum McMaster University Hamilton Ontario Canada

## Abstract

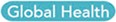

## Ebola was a Teachable Moment

On December 29, 2015, when many people were preparing to welcome the New Year of the Gregorian calendar, the World Health Organization (WHO) announced its own cause for celebration: Ebola was gone from Guinea and there had not been a single new case worldwide since November (WHO, 2016a). WHO declared the outbreak had ended in January, after 42 days of zero cases. New isolated cases of Ebola have popped up since then, but everyone agrees the worst is behind us.

West Africa's Ebola outbreak was the health news story of 2014 and 2015. Identified in March 2014 and declared an emergency in August 2014, the virus spread quickly in a region that had never before experienced it. The result was harrowing. At least 28,646 people contracted the virus, and 11,323 people died (WHO, 2016c). Even worse is how the outbreak eviscerated the health systems and economies of Guinea, Liberia, and Sierra Leone – three of the world's poorest countries – whose 22 million people will be affected for decades to come.

This Ebola outbreak was a scary drama for people everywhere. I still remember the fear in my compatriots' voices who called in to a Canadian Broadcast Corporation radio programme I was chairing. One man expressed his concern about riding Toronto's subway. A woman revealed her apprehension of associating with African‐Canadians. Others predicted doom unless every country immediately closed its borders.

Of course these fears were never merited, but the greatest Ebola fear seems to be at the cusp of realization: that we will not learn from this Ebola experience and that we will not address the underlying global institutional failures that allowed it to occur in the first place. What a tragedy it would be if we need a far worse pandemic than Ebola, one that kills many more people and wreaks even greater havoc, to finally motivate the global institutional changes that are urgently required.

## Three Simple Lessons Learned

Of course, we have learned *something* from this Ebola outbreak. We have actually learned many things – captured well by the numerous “Ebola lessons learned” exercises that have been undertaken by the United Nations (UN, 2016), WHO (WHO, 2015a, 2015b), independent panels (Moon et al., 2015; National Academy of Medicine, 2016), and individual academics (Kamradt‐Scott, 2016). At least three simple lessons stand out.

The first lesson is how properly funded public health interventions can stop the spread of viruses in their tracks, even in this age of hypermobility and transcontinental travel and even after a virus has spread internationally. Hard work remains ahead to maintain heightened disease surveillance and rebuild what was destroyed, but the worst is now behind us thanks to the solidarity of West African citizens and the world's public health professionals.

The second lesson is the need to invest in research and development for new technologies and strategies that can help make us better prepared for future outbreaks of new and neglected diseases (Hoffman and Røttingen, 2012). One important contribution in this particular outbreak response was the development and testing of Ebola vaccines (Henao‐Restrepo et al., 2015). We are all safer as a result.

The third lesson involves the weakness of our global health agencies and how desperately they must be reformed. Many of us global health researchers have been ringing alarm bells for years – flagging WHO's weaknesses (Hoffman and Røttingen, 2014), the unenforceability of the *International Health Regulations* that legally govern countries' responses to pandemics (Hoffman, 2014), insufficient support for boosting national disease outbreak response capacities (Edge et al., 2013), and destructive competition among global health agencies (Hoffman, 2010). But now, the names and failings of these global health institutions have been splashed across the front pages of the world's leading newspapers and on prime‐time television. Reforming WHO and the *International Health Regulations*, for example, has attracted political attention at the highest levels, including G7 heads of government and the United Nations Security Council. Ordinary citizens have discussed these agencies' failings in public forums, on the radio, and at their kitchen tables.

Learning lessons is great, but such lessons will only help the world and honour the 11,323 lives lost in this Ebola outbreak if we actually act upon them. So far that has not happened in a sufficiently meaningful way. Certainly, we are now seeing the start of some reforms. For example, the World Bank created a Pandemic Emergency Financing Facility and the US‐led Global Health Security Agenda has assumed a stronger leadership role in promoting national pandemic response capacities. WHO has been particularly busy with lots of processes that will hopefully lead to tangible improvements. The agency has consolidated its health emergency response staff into a single unified programme, formed an independent oversight committee to guide it, created a contingency fund for emergency, and started quality assurance assessments of emergency medical teams (WHO, 2016b).

But despite these nascent efforts over the past 2 years since the Ebola outbreak started, public health capacity remains insufficient, disincentives for funding neglected disease research have not been resolved, and our global health agencies mostly remain as they have long been. Additional reforms are currently being debated, but progress is glacial in pace and time is running out. My own research shows that major disease outbreaks like Ebola can create a 3‐year policy window to make change before attention shifts elsewhere. More than 2 years have passed; we have less than 1 year left to go.

## More Action is Needed Urgently

All of this matters greatly to the health, well‐being, and future prosperity of our world, and each of us, our civil society organizations, companies and governments have important roles to play in making the world a safer place. For example, national governments can fund the Pandemic Emergency Financing Facility, encourage changes to WHO governance, and clear the legal and regulatory barriers that diminished humanitarian organizations' ability to quickly respond (Dutchak et al., 2016). WHO's member states can add tough sanctions to the *International Health Regulations*, both because enforcement mechanisms work (Hoffman and Ottersen, 2015) and so that the 40+ countries that imposed Ebola travel restrictions against West Africans can redeem themselves of violating this legally binding (albeit toothless) treaty (WHO, 2015c).

The reality is that without global institutional innovation, we will continue to be ever more threatened by disease outbreaks like Ebola. Our world is getting smaller, with people travelling farther and more often than ever before, carrying potentially dangerous bacteria and viruses wherever we go. Wealthier countries are particularly vulnerable as the greatest source and destination of global travellers, which gives them special moral responsibility (and self‐interest) to champion multilateral efforts to address global health threats like pandemics.

So how many people must a pandemic kill before the world actually addresses the underlying global institutional failures that allow them to occur? I do not know. But 2016 will show us whether 11,323 deaths were enough.
